# Chrononutrition during Pregnancy: A Review on Maternal Night-Time Eating

**DOI:** 10.3390/nu12092783

**Published:** 2020-09-11

**Authors:** See Ling Loy, Rachael Si Xuan Loo, Keith M. Godfrey, Yap-Seng Chong, Lynette Pei-Chi Shek, Kok Hian Tan, Mary Foong-Fong Chong, Jerry Kok Yen Chan, Fabian Yap

**Affiliations:** 1 Department of Reproductive Medicine, KK Women’s and Children’s Hospital, 100 Bukit Timah Road, Singapore 229899, Singapore; jerrychan@duke-nus.edu.sg; 2 Duke-NUS Medical School, 8 College Road, Singapore 169857, Singapore; tan.kok.hian@singhealth.com.sg; 3 Singapore Institute for Clinical Sciences, Agency for Science, Technology and Research (A*STAR), 30 Medical Drive, Singapore 117609, Singapore; obgcys@nus.edu.sg (Y.-S.C.); lynette_shek@nuhs.edu.sg (L.P.-C.S.); mary_chong@nus.edu.sg (M.F.-F.C.); 4 Department of Paediatrics, KK Women’s and Children’s Hospital, 100 Bukit Timah Road, Singapore 229899, Singapore; rachael.loo.s.x@kkh.com.sg; 5 Medical Research Council Lifecourse Epidemiology Unit, University of Southampton, Southampton SO16 6YD, UK; kmg@mrc.soton.ac.uk; 6 National Institute for Health Research Southampton Biomedical Research Centre, University of Southampton and University Hospital Southampton National Health Service Foundation Trust, Southampton SO16 6YD, UK; 7 Yong Loo Lin School of Medicine, National University of Singapore, National University Health System, Singapore 119228, Singapore; 8 Department of Paediatrics, Yong Loo Lin School of Medicine, National University of Singapore, National University Health System, Singapore 119228, Singapore; 9 Khoo Teck Puat-National University Children’s Medical Institute, National University Hospital, National University Health System, Singapore 119074, Singapore; 10 Department of Maternal Fetal Medicine, KK Women’s and Children’s Hospital, Singapore 229899, Singapore; 11 Saw Swee Hock School of Public Health, National University of Singapore, National University Health System, Singapore 117549, Singapore; 12 Lee Kong Chian School of Medicine, Nanyang Technological University, 11 Mandalay Road, Singapore 308232, Singapore

**Keywords:** chrononutrition, circadian rhythm, lifestyle behavior, night eating, pregnancy

## Abstract

Evidence from women working night shifts during pregnancy indicates that circadian rhythm disruption has the potential to adversely influence pregnancy outcomes. In the general population, chronodisruption with the potential to affect pregnancy outcomes may also be seen in those with high energy intakes in the evening or at night. However, maternal night eating during pregnancy remains understudied. This narrative review provides an overview of the prevalence, contributing factors, nutritional aspects and health implications of night eating during pregnancy. We derived evidence based on cross-sectional studies and longitudinal cohorts. Overall, night eating is common during pregnancy, with the estimated prevalence in different populations ranging from 15% to 45%. The modern lifestyle and the presence of pregnancy symptoms contribute to night eating during pregnancy, which is likely to coexist and may interact with multiple undesirable lifestyle behaviors. Unfavorable nutritional characteristics associated with night eating have the potential to induce aberrant circadian rhythms in pregnant women, resulting in adverse metabolic and pregnancy outcomes. More research, particularly intervention studies, are needed to provide more definite information on the implications of night eating for mother-offspring health.

## 1. Introduction

The timing of eating has become an important area of research given the increasing recognition of food intake as a zietgeber (time cue) for the circadian system of humans [1]. Eating food at times that contradict body’s natural circadian rhythms, such as eating during the inactive/sleep phase, has been shown to entrain the expression of clock genes in most peripheral tissues, e.g., liver, pancreas, skeletal muscle, adipose tissue, leading to misalignment between internal circadian rhythms [2,3]. Consequently, there is a potential adverse effect on metabolic physiology, increasing the risk of diabetes, obesity, cardiovascular disease and possibly even cancer [4,5,6]. Thus, desynchronization between the light-entrainable central clock in the brain and food-entrainable peripheral clocks in tissues appears to be disadvantageous for metabolism [7]. This refers to the concept of chrononutrition—the study of food’s impacts on metabolism via the circadian clock system [8]. Chrononutrition reflects the basic concept that not only food quantity and quality, but also food timing is critical for the well-being of an individual [9].

Maternal adaption in pregnancy induces changes in circadian rhythms [10] with marked alterations in the expression of circadian clock genes [11,12]. More specifically, changes in maternal peripheral clock gene expressions across pregnancy drive downstream shifts in circadian expression of certain metabolic genes, such as glucoregulatory genes Pck1, G6Pase and Gk to support healthy pregnancy [12]. This indicates that when circadian rhythms during pregnancy are disrupted, there is a potential for putting women at risk of developing metabolic disorders and adverse pregnancy outcomes. There is established evidence that pregnant night-shift workers are at risk of miscarriage, prematurity, low birth weight and hypertensive disorders [13,14]. These findings have relevance to not only pregnant night-shift workers, but may also apply to the general pregnant population who consume high energy intakes in the evening or at night with potential chronodisruption. Night-time is a period when the body is naturally primed for rest in humans. Among pregnant women, the prevalence and clinical significance of night eating are poorly understood.

In this review, we focus on epidemiological and clinical evidence studying night eating of pregnant women, and its link with pregnancy outcomes. Specifically, we (1) describe the assessment methods and prevalence of night eating during pregnancy; (2) discuss the potential reasons, characteristics and nutritional aspects of maternal night eating; (3) summarize the associations of night eating with maternal metabolic health and pregnancy outcomes; and (4) conclude by outlining potential areas for future investigation. We include studies of night eating in adult populations to compare relevant findings in pregnant women.

## 2. Assessment and Definition of Night Eating

The dietary assessment methods and definitions of night eating vary widely [15]. Herein, we describe published reports that used either quantitative (e.g., food record/diary, 24-h recall) or qualitative (e.g., self-estimation of energy intake, questionnaire) dietary assessment methods to assess night eating during pregnancy (Table 1).

Only three research groups (United States, Singapore and Brazil) were identified as studying maternal night eating using food diaries or recalls [16,17,18]. In a study of African American women, a 2 day food diary was used to assess maternal night-time energy intake in the third trimester of pregnancy, as determined by an average of total daily energy intake (TDEI) between 8:00 p.m. and 5:59 a.m. [16]. However, the basis for focusing on the period of 8:00 p.m.–5:59 a.m. is unclear. The Growing Up in Singapore Towards healthy Outcomes (GUSTO) study in Singapore used a single 24-h recall (validated using a 3-day food diary in a subset) to derive the maternal night eating variable in the second trimester of pregnancy [17,23]. Night-time energy intake was based on TDEI between 7:00 p.m. and 6:59 a.m., the period aligns with local sunset and sunrise times throughout the year in Singapore. Women were defined as predominantly night eaters if consuming more than 50% of TDEI between 7:00 p.m. and 6:59 a.m. More recently, Gontijio and colleagues from Brazil determined night-time energy intake based on the average of TDEI between 7:00 p.m. and 5:59 a.m., using three non-consecutive 24 h recalls (one weekend and two weekdays) at each trimester of pregnancy [18]. Women were classified into higher night-time intake if consuming night-time energy above the median of the population for at least two trimesters. The period of 7:00 p.m.–5:59 a.m. was determined by the annual average of nightfall time frame.

Other studies have used questionnaires, either as a set of questions or a single question, to assess maternal night eating. The Norwegian Mother and Child Cohort Study (MoBa) asked questions on the weekly frequencies of eight meals/snacks (breakfast, morning snack, lunch, afternoon snack, dinner, evening snack, supper and night meal). Based on the frequencies, an evening meal pattern, as characterized by supper and night meals, was derived using principal component factor analysis [19]. It should be noted that individuals consuming high energy at night may not necessarily have features of night eating syndrome (NES), an eating disorder involving morning anorexia, evening hyperphagia and/or nocturnal ingestion and insomnia [24]. Dietary behavior of NES patients were considered beyond the scope of ‘normal’ eating [15]. In the following section, we include the few NES studies to compare its prevalence rate with night eating. Thus far, we have identified two studies involving Turkish and African American pregnant women that examined maternal NES [20,21]. These studies used the Night Eating Questionnaire (NEQ), a validated 14-item questionnaire [25] to examine NES in pregnant women. In the NEQ, evening hyperphagia is assessed by asking participants to estimate the proportion of TDEI after dinner, with the cut-off of 25% TDEI increment for each option in a 5-point Likert scale [25]. A study from Poland evaluated maternal night eating by asking whether women woke up at night and ate [22].

## 3. Prevalence of Maternal Night Eating

**Figure 1:**
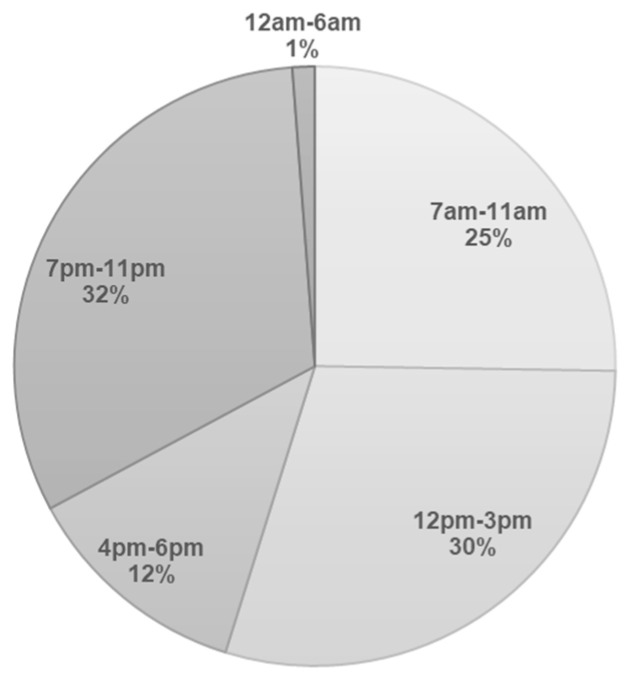
Distribution of total daily energy intake (%) by 24 h day among pregnant women at 26–28 weeks gestation from the GUSTO study (*n* = 1095). GUSTO, Growing Up in Singapore Towards healthy Outcomes.

In adults, there is a global trend towards increased energy intake later in the day [26]. Owing to the possible change in dietary habits during pregnancy [27], it is unclear whether there are similar trends of delayed temporal meal distribution in pregnant women. As the available published information about maternal meal timing throughout the day is sparse, we drew on data from the GUSTO cohort comprising Asian pregnant women (*n* = 1095) at 26–28 weeks gestation with the aim of understanding maternal temporal distribution of TDEI over the 24 h in Singapore. We observed a lower proportion of TDEI in the morning than in the afternoon and at night. Specifically, 25% of TDEI were consumed in the morning (7:00 a.m.–11:00 a.m.), 30% of TDEI were consumed in the afternoon (12:00 p.m.–3:00 p.m.) and 32% of TDEI were consumed at night (7:00 p.m.–11:00 p.m.) (Figure 1). Of these women, 15% consumed more than 50% of their TDEI during the period of 7:00 p.m.–6:59 a.m. [17]. In the study by Gontijio et al. [18] involving 100 pregnant women in Brazil, 45% of them had high energy intake more than the medians between 7:00 p.m. and 5:59 a.m. for at least two trimesters.

A study of African American pregnant women (*n* = 40) at 32–34 weeks gestation reported that an average of 25% of TDEI was consumed between 8:00 p.m. and 5:59 a.m. [16]. An earlier study indicated that 32% of African American pregnant women (*n* = 120) at 14–24 weeks gestation had more than 25% of TDEI after dinner time [20]. This suggests a high prevalence of evening hyperphagia, but the high intake of night-time energy was unlikely to be explained by the NES, as only 4% women met the criteria for NES using the NEQ [20]. For comparison, we specifically analyzed energy intake of women from the GUSTO study after the period of common dinner time (7:00 p.m.–8:00 p.m.). We found that 22% (235/1095) of this group of pregnant women in Singapore consumed more than 25% of TDEI after 9:00 p.m. In Poland (*n* = 266), 23% of women at 28–41 weeks gestation reported waking up at night and eating [22]. Although a high prevalence of nocturnal food intake was suggested, there was no examination of night-time energy intake. In Turkey (*n* = 148), 12% women at 28–38 weeks gestation were screened as positive for NES using the NEQ [21].

Maternal night eating is considered common during pregnancy. The prevalence of night eating is expected to be much higher than NES, ranging from 15% to 45% during pregnancy depending on definitions used [17,18,20,22]. Most studies have been cross-sectional and trimester-specific, with little known about how the daily energy distribution and night eating behaviors change across trimesters and impact on health. The question has been partly addressed by a prospective cohort studying women from the first until the third trimesters; this reported that the temporal distribution of eating pattern including meal timing and frequency remained constant throughout the gestation trimesters [18]. However, the small sample size (*n* = 100) might have restricted the generalizability of findings to wider and other pregnant populations.

## 4. Potential Reasons for Maternal Night Eating

It is unclear whether maternal night eating is a behavior established before conceiving, or exaggerated or exhibited only during pregnancy. This information is important to determine the target groups and time-point of interventions addressing night eating (i.e., preconception or trimester specific during pregnancy). Understanding potential reasons for maternal night eating during pregnancy might provide some clues on this aspect, and useful baseline information in developing intervention strategies.

Lifestyle habits and time pressure have been frequently cited as contributors to unhealthy eating, including night eating [28]. Past studies have associated long working hours and shift work with irregular meal timing. Escoto et al. [29] studied the relationship between the number of working hours per week and time-related beliefs to healthy eating among 2287 American adults. The study reported that individuals with longer working hours (>40 h per week) often pay little attention to nutritional balance and have late dinners [29]. A study in South Korea involving 340 nurses found that those with rotating night-shift schedules were more often engaged in breakfast skipping and late night snacking, as compared to nurses without night-shifts [30]. Thus, it is expected that the same scenario can occur in working women who are pregnant. However, compared to before pregnancy, women may be more likely to practice night eating after becoming pregnant due to the presence of pregnancy symptoms and discomforts [31], which could disrupt their eating patterns [27].

To our knowledge, only one study to date has explored the reasons for night eating among pregnant women. Based on a qualitative survey involving 18 pregnant African American women (27–38 weeks gestation) who regularly ate between 8:00 p.m. and 6:00 a.m., altered sleep schedules, hunger, thirst, nausea, fetal movement and personal choice were the common reasons for night eating [32]. These women reported having difficulty sleeping at night, which caused them to wake up later in the morning and delay their entire meal timing throughout the day [32]. This is supported by a study conducted in 266 Polish women (28–41 weeks gestation), showing that night eating was associated with insomnia during pregnancy, although any causal-effect relationship is unclear [22]. Forty percent of women who developed insomnia during pregnancy reported constantly waking up at night to eat [22]. Since insomnia occurs most frequently during the third trimester of pregnancy [33,34], this may be accompanied with more frequent night eating.

In a qualitative survey by Kroeger et al. [32], a number of women reported constantly feeling hungry during pregnancy. Some of them regularly woke up at a specific time during the night to eat and drink due to hunger and thirst. However, the role of appetite in night waking behavior during pregnancy is uncertain. It has been proposed that such a phenomenon may plausibly be due to impaired fat oxidation in these women [32]. Fatty acids are the main energy source during overnight fasting [35,36]. There is evidence that individuals who engage in night eating have decreased fat oxidation [37,38,39]. Although fat oxidation was found to be downregulated among obese individuals [40] and those who are prone to obesity [41], little is known about whether there is downregulation of fat oxidation during pregnancy, which could contribute to hunger at night.

It has been documented that pregnant women tend to experience nausea in the morning, causing them to eat less during the day but more at night [32]. This was supported by a study in Brazil following pregnant women from 4 to 37 weeks gestation, showing breakfast skipping was more prevalent in night eaters than day eaters throughout pregnancy [18]. Another study involving Norwegian pregnant women at 17–22 weeks gestation reported that nausea was positively associated with an evening meal pattern tendency [19]. In addition to nausea, low appetite in the morning might also cause women to delay their temporal distribution of food intake. In a clinical trial examining circadian influences on appetite among 12 healthy adults, the circadian rhythm in hunger reached a trough at 8 a.m., indicating appetite was clock-controlled and reached at its lowest in the morning [42].

Night-time fetal movement is common during the third trimester of pregnancy; a few studies have demonstrated that fetal movement increased along the day, with peak activity between 9:00 p.m. and 1:00 a.m. [43,44]. Some pregnant women have interpreted fetal movement as a fetal demand for food, prompting them to wake up and eat [32,45].

On top of pregnancy symptoms and physiological reasons, night eating during pregnancy has been reported to be influenced by personal preference and psychosocial factors [32]. For personal preference, it is consistent with the Theory of Planned Behavior, indicating that individuals often hold a belief or attitude about eating whenever they want to rather than adhering to a fixed eating schedule [46]. This applies to food craving, which is common during pregnancy [27], and craving frequency has been associated with increased night snacking [47]. The influence of other members in the household was cited as the primary psychosocial influence on night eating [32]. This suggests that the likelihood of engaging in night eating is related to an individual’s beliefs and household environment.

## 5. Characteristics Associated with Maternal Night Eating

Aside from occupation, as previously described (working hours and shift works), variations in other socio-demographic factors may be partly responsible for the differences in food timing. The younger adult population tend to engage in night eating [48]. In the MoBa study, younger Norwegian women (<35 vs. ≥35 years) had a higher adherence to evening meal pattern during pregnancy [19]. In addition, a strong correlation with education was observed, with shorter education duration (≤12 years) being more common in pregnant women with a higher evening meal pattern tendency [19]. Similarly, the GUSTO study in Singapore found that lower education attainment was more common in pregnant night eaters than day eaters [17].

The night eating pattern during pregnancy may be vary by pre- or early-pregnancy BMI status. Chandler-Laney et al. [16] found that obese African American women (early-pregnancy BMI ≥ 30 kg/m^2^) consumed higher night-time energy in their third trimester than those with normal-weight early-pregnancy BMI (<25 kg/m^2^). Questions arise as to whether pre-pregnancy obesity blunts feeding/fasting cycles and induces high night-time energy intake during pregnancy [49], or whether night eating in late pregnancy is persistently observed before pregnancy and may dysregulate metabolism and lead to high pre-pregnancy BMI [5,50]. In contrast, Englund-Ögge et al. [19] reported that Norwegian women who were underweight (BMI < 18.5 kg/m^2^) prior to pregnancy had a greater evening meal pattern adherence in their second trimester. A few studies observed no association between pre- or early-pregnancy BMI and night eating during pregnancy [17,18].

In Brazil, women with night eating were more likely to skip breakfast and be physically inactive during pregnancy than those with day eating [18]. In Norway, women with higher adherence to evening meal pattern smoked more frequently during pregnancy [19]. In Singapore, pregnant night eaters had higher anxiety scores than day eaters [51]. A population study in Korea (*n* = 31,690) revealed that women, but not men, who engaged in night eating were more likely to develop depression and depressive symptoms [52]. Taken together, these studies indicate the likely co-existence of multiple undesirable lifestyle behaviors in women with night eating during pregnancy.

Night eating patterns have been linked with chronotypes, where evening-type individuals are likely to engage in night eating. Evening preference was shown to be associated with frequent night meals [53] and high caloric intake before bedtime [54]. As evidenced by a population-based study of more than 1800 adults, those with later chronotypes consumed a higher caloric intake after 8:00 p.m. compared with earlier chronotypes [55]. Similarly, during pregnancy, women with night eating were more likely to have an evening chronotype [18].

Understanding characteristics associated with maternal night eating in non-pregnant populations could provide insights into factors to be taken into account when studying night eating during pregnancy. Furthermore, some of the factors may interact with night eating to induce deleterious effects on health. For example, among 60,800 Japanese adults, those with both night eating and breakfast skipping had an increased risk of metabolic syndrome and proteinuria, but no associations were observed when night eating and breakfast skipping were examined independently [56]. Further studies in this area of pregnancy are required.

## 6. Nutritional Aspects of Night Eating

A general rhythm of macronutrient intake has been shown throughout the day, where morning intake tends to be relatively high in carbohydrates, midday intake tends to be relatively high in protein and night-time intake tends to be relatively high in fat [57,58]. A tendency to select foods rich in carbohydrate for consumption during the night has also been reported [59]. Understanding the nutrient intakes of night eaters may help to elucidate potential mechanisms involved, examine effects of night eating on health, and to establish nutritional recommendations for pregnant women.

In Singapore, compared to pregnant day eaters, night eaters had a higher intake of total dietary fat, but a lower intake of total dietary carbohydrate [17]. Specifically, higher night-time intake of dietary fat was observed in this group of night eaters than day eaters (36% vs. 28%; *p* < 0.001). It has been shown that a high fat diet could blunt feeding/fasting cycles, increasing the proportion of energy intake during the night and subsequently dampening circadian rhythms in clock genes [49]. Another study from Brazil reported that both total dietary fat and carbohydrate intakes were lower in pregnant night eaters than day eaters [18]. However, these night eaters tended to consume more dietary carbohydrates at night [18]. Despite differences in macronutrient composition, both studies reported that night eaters consumed fewer TDEI than day eaters during pregnancy [17,18]. In contrast, a study from Norway indicated that pregnant women with higher adherence to an evening meal pattern consumed a higher TDEI [19]. Meanwhile, they were also found to have a higher dietary glycemic load [19]. Altogether, these studies suggest that other than TDEI, night eating was associated with macronutrient profiles, including quality of carbohydrate.

In terms of micronutrients, Gontijio and colleagues [18] demonstrated that increased night-time energy intake was associated with lower intakes of dietary riboflavin, calcium and iron during pregnancy. Given that night eaters were more likely to be evening chronotypes [60], the authors further showed that pregnant women with an eveningness tendency had lower diet quality and fewer fruit intake, than those with a morningness tendency [61]. Similarly, the GUSTO research group observed that pregnant night eaters consumed lower amounts of total fruit and whole grains, and had a lower diet quality than day eaters (Table 2). The diet quality was evaluated by the Healthy Eating Index for pregnant women in Singapore [62]. Further investigation of circulating micronutrient concentrations found that plasma folate and vitamin D levels were lower in night eaters than in day eaters (Table 3). This is consistent with the findings of low levels of multiple circulating nutrients among pregnant breakfast skippers [63], who were found to frequently engage in night eating [18]. For vitamin D, low plasma 25-hydroxyvitamin D which could be a surrogate measure of retinal sunlight exposure has been suggested to disrupt circadian rhythms via dysregulating the central clock [64]. Together, these studies suggested that night eating may be associated with undesirable micronutrient profiles.

Although studies are too limited to make firm conclusions regarding nutritional characteristics related to night eating, the available information points towards maternal night eating being generally associated with less healthy dietary intake, low diet quality and reductions in circulating levels of certain micronutrients during pregnancy. This is supported by studies in teenagers and adults, which have consistently reported that individuals with a delayed temporal distribution of food intake (i.e., night-eaters, late-eaters, an evening chronotype, night-shift workers) were mostly engaged in unhealthy eating behavior with poor diet quality [59,60,65]. Overall, it seems that diet composition among night eaters is an important aspect to consider when studying night eating and health implications. Beyond TDEI, the undesirable nutritional characteristics associated with night eating may be one of the potential factors implicated in adverse maternal health during pregnancy. The type and amount of macro and micronutrients play roles as modulators of human metabolic health via circadian clock regulation [7]. This is evidenced by the ability of diet composition, such as fat, fatty acids, sugar and vitamins, to function as zeitgebers for the circadian clock [7].

## 7. Night Eating and Maternal Health Outcomes

There is evidence of impaired glucose metabolism in response to night eating among pregnant women [16,17], which is likely attributable to relatively low insulin sensitivity during this time [66]. However, a weight-dependent effect was observed. A study by Chandler-Laney et al. [16] showed that independent of daytime energy intake, increased night-time energy intake was associated with reduced dynamic β-cell response in the obese pregnant women (pre-pregnancy BMI ≥ 30 kg/m^2^), but not in those who were of a normal weight (pre-pregnancy BMI < 25 kg/m^2^). In contrast, a later study by Loy et al. [17] reported that independent of TDEI, pregnant night eaters who were lean (pre-pregnancy BMI < 23 kg/m^2^) had an increased fasting plasma glucose, but this association was not found in those who were overweight/obese (pre-pregnancy BMI ≥ 23 kg/m^2^). The authors proposed that this discrepancy may be due to the marked suppression of insulin sensitivity in the morning among overweight/obese individuals, leading to failure detection for a further reduction in insulin sensitivity [17]. In the aforementioned study [16], when further examination of night-time carbohydrate intake was performed, independent of TDEI, a high carbohydrate intake at night instead of during the day was associated with reduced glucose tolerance and lowered insulin secretion in obese pregnant women. This finding is supported by an earlier study which revealed that night snacking of carbohydrate-rich foods was more often observed in women developing gestational diabetes mellitus (GDM) than those with normal glucose tolerance [67]. Observations in pregnant women are in agreement with evidence in adults, indicating that food timing and the amount of dietary carbohydrate could affect glucose metabolism [59]. Indeed, dietary carbohydrate composition (e.g., highly vs. poorly digestible carbohydrate) has been shown to heavily influence the clock in regulating glucose homeostasis [7]. Taken together, maternal night-time energy intake and specifically, the amount and type of night-time carbohydrate intake have the potential to affect glucose tolerance and β-cell function during pregnancy, and the risk of GDM development.

A prospective cohort study reported that compared to pregnant women with lower night-time energy intake, those with higher night-time energy intake had an adverse pattern of gestational weight gain (GWG) in the third trimester, independent of pre-pregnancy BMI [18]. Of note, there were no differences in TDEI and macronutrient intake in the third trimester between these groups of pregnant women [18]. Another prospective cohort study further demonstrated that night eating during pregnancy was associated with greater weight retention of at least 5 kg at 18 months postpartum, independent of TDEI and early pregnancy BMI [68]. GDM, GWG and breastfeeding practice after delivery did not alter this association [68]. Based on this observation, the authors suggested the possibility of persistent night eating behavior beyond pregnancy, with implications for long-term obesity risk [68]. These findings are consistent with a meta-analysis of observational studies in adults, reporting an association between greater night-time energy intake and higher BMI [15]. Given that night time is a period with delayed gastric emptying [69], decreased thermic effect of food and reduced resting metabolic rate [70], consuming a large amount of energy during this period may be detrimental to metabolic processes during pregnancy by dysregulating circadian rhythms, disrupting hormone secretion and altering gut microbiome [2,71].

Aside from metabolic implications, maternal night eating has been associated with the duration of gestation. Pregnant night eaters in the second trimester were reported to exhibit a shorter gestation and a greater likelihood of delivering preterm (<37 weeks gestation), independent of TDEI, sociodemographic and lifestyle factors [51]. This association was not altered by bedtime or glycemic measures during pregnancy [51]. The study provided new evidence on the role of night eating in preterm delivery. Indeed, this finding is supported by the Nurses’ Health Study, which demonstrated an increased risk of preterm birth in nurses working night-shifts, most likely explained by circadian disruption [72]. It has been reported that consuming foods at night may suppress melatonin [73] due to circadian misalignment [3], leading to dysregulation of oxytocin, uterine contractility and birth timing [74]. Although the shift or delay in meal timing during the rest phase is less severe in night eaters compared to shift workers, pregnant women with night eating may remain exposed to a milder form of chronodisruption that contributes to preterm birth. In contrast, evening meal pattern during mid-pregnancy as assessed in the MoBa study was not associated with preterm birth; however, the study did not evaluate night-time energy intake [19].

## 8. Concluding Perspectives

To obtain a better evaluation and understanding of maternal night eating, the use of multiple-day food diaries or 24 h recalls across different trimesters of pregnancy is an ideal approach. In addition to being more reliable, it enables the evaluation of night eating behavior changes through pregnancy. Importantly, using a food diary or recall allows flexibility of using different time frames and different energy intake cut-offs to define night eating and night eaters according to the local context, e.g., nightfall period and dinner time, as geographical and cultural differences are present in the temporal distribution of eating. This method hinders data harmonization for comparing findings, but the key is to understand how a certain proportion of TDEI and diet composition during a specific period of the night within a population could impact on health. This facilitates the design and development of dietary intervention based on meal timing which is socially and culturally appropriate.

**Figure 2:**
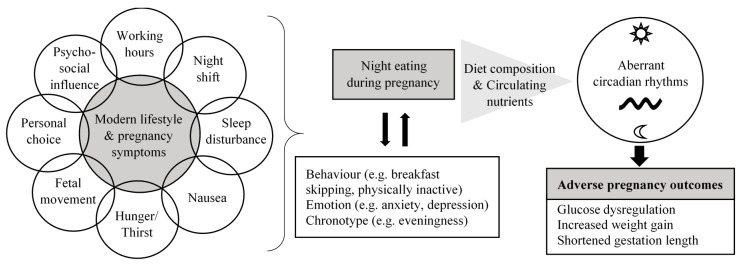
Potential contributors, associated characteristics and effects of night eating during pregnancy. Modern lifestyle and the presence of pregnancy symptoms contribute to night eating during pregnancy, which coexists and may interact with multiple undesirable lifestyle behaviors. Unfavorable nutritional characteristics associated with night eating have the potential to induce aberrant circadian rhythms of pregnant women, resulting in adverse pregnancy outcomes.

The current review provides an overview of the potential contributing factors, nutritional characteristics and health effects of night eating during pregnancy based on evidence from observational studies (Figure 2). Targeting nutrition as a strategy to optimize pregnancy outcomes is an important research agenda for scientists and dietitians/nutritionists. Timing of energy intake and diet composition appear as potential novel approaches to address metabolic complications during pregnancy and adverse birth outcomes. Based on the studies discussed, though with limited numbers, a high night-time energy intake and its nutritional profile may play a role in contributing to impaired energy and glucose metabolism, and reproductive hormone disruption during pregnancy. In other words, it seems that managing the amount of energy intake and food choice during the night may offer part of the solution to reducing adverse pregnancy outcomes. Intervention trials are warranted to provide more definitive information in this field. Food types, attitude, behavioral components (e.g., lifestyle and chronotype) and pregnancy symptoms relating to night eating are important aspects to be considered in the design of intervention that aims at addressing night eating. This will help to facilitate adherence and to better predict the effectiveness of a given intervention by accounting for these factors in the analysis. If the results are promising, management strategies for night eating should be addressed in nutrition guidelines and counselling for pregnant women.

To have a more thorough understanding of how night eating relates to health, both evening meals and nocturnal snacks should be analyzed separately due to the potential variation in metabolic implications. A snack, which has been defined as an event not motivated by physiological hunger, but elicited by an external non-physiological stimulus, has been proposed to exhibit different energy substrate utilization from taking a meal, which is triggered by hunger [75]. Increasing evidence suggests that negative metabolic implications may not occur if the bedtime snack is low in calories and rich in certain nutrients such as protein [76]. In addition, in studying food timing, components including meal (ir)regularity (meal skipping/delaying), eating frequency (number of meals and snacks) and fasting interval (duration of daytime or night-time fasting, spacing of eating) should be taken into account as they are inter-connected to each other. In a recent large-scale clinical trial using machine-learning to predict human postprandial responses to food intake, both meal timing and periods of fasting have been proposed as offering a large potential for improving the prediction of postprandial responses linked with cardiometabolic disease [77,78].

Nevertheless, most aforementioned components have been scarcely explored in pregnant women. A few epidemiological studies have reported that pregnant meal skippers may be at risk of preterm delivery [19,79,80,81]. In particular, breakfast skipping in early pregnancy was associated with offspring obesity development through the first 12 years of life [82,83]. However, it is not known whether the observed outcome was contributed by breakfast skipping or because of night eating, given the positive association between these two variables [18]. This suggests a need for future studies to take into account night-time energy intake to determine the relative benefit of breakfast eating compared to night eating. In addition, compared to dinner skipping, whether breakfast skipping imposes a more detrimental health effect remains an unanswered question. This is built on the basis of hypothesized differential phase effects (day and night) of fasting period according to natural circadian rhythms on metabolic regulation.

As most existing studies have focused on a specific trimester for dietary assessment, prospective cohorts are needed that assess maternal diet using multiple food diaries or recalls across trimesters, and even during the preconception phase. This will allow the investigation of meal pattern changes and specific windows when circadian eating may have started to impose a detrimental effect on pregnancy outcomes. Advanced technologies, such as using mobile phone apps to record diet and food images with time-stamps, and the wearing of continuous recording devices to measure 24-h blood glucose and activity, will provide valuable information to aid understanding of how temporal eating patterns influence physiological processes and health. Importantly, investigations on mechanistic pathways underlying night eating and maternal-fetal health outcomes, immediately and long-term, are required. In pregnant rodents, disrupted food timing (eating during the day for nocturnal animals) has been shown to cause aberrant circadian rhythmicity in both the mother and fetus [84], and could potentially modify microbiota profiles in ways that lead to metabolic disorders [71], but the translation of these mechanisms to pregnant women has yet to be performed.

## Figures and Tables

**Table 1:** Studies assessing maternal night eating during pregnancy.

Reference	Year of Publication	Country	Diet Assessment Time-Point	Sample Size	Gestation Weeks	Method of Dietary Assessment	Definition of Night-Time Eating
Chandler-Laney et al. [16]	2015	United States (African American)	Single	40	32–34	2-day food diary	Higher TDEI during 8:00 p.m.–5:59 a.m.
Loy et al. [17]	2016	Singapore	Single	985	26–28	Single 24-h recall	More than 50% of TDEI during 7:00 p.m.–6:59 a.m.
Gontijio et al. [18]	2020	Brazil	Multiple	100	4–12; 20–26; 30–37	Three 24-h recalls per trimester	Above the median of TDEI during 7:00 p.m.–5:59 a.m.
Englund-Ögge et al. [19]	2017	Norway	Single	65,487	17–22	Questions asking frequency of eating events	Evening meal pattern as characterized by intake of supper and night meals
Allison et al. [20]	2012	United States (African American)	Single	120	14–24	Night Eating Questionnaire	Night Eating Syndrome
Damla Deniz et al. [21]	2019	Turkey	Single	148	28–38	Night Eating Questionnaire	Night Eating Syndrome
Wołyńczyk-Gmaj et al. [22]	2017	Poland	Single	266	28–41	Single question	Awake at night and eat

TDEI, total daily energy intake.

**Table 2:** Comparisons of individual components of HEI-SG between day eaters and night eaters (*n* = 974) at 26–28 weeks gestation.

Component	Day Eaters (*n* = 834)	Night Eaters (*n* = 140)	
	Mean ± SE	Mean ± SE	*p*-Value
Total fruit	2.4 ± 0.1	1.7 ± 0.3	0.045
Whole fruit	2.2 ± 0.1	1.6 ± 0.3	0.091
Total vegetables	2.4 ± 0.1	2.2 ± 0.2	0.535
Dark green leafy and orange vegetables	2.0 ± 0.1	1.9 ± 0.3	0.898
Total rice and alternatives	8.8 ± 0.1	8.5 ± 0.3	0.312
Whole grains	1.2 ± 0.1	0.5 ± 0.4	0.047
Total protein foods	7.7 ± 0.1	8.1 ± 0.4	0.325
Dairy	5.6 ± 0.2	5.1 ± 0.6	0.448
Total fat	6.4 ± 0.2	5.8 ± 0.5	0.306
Saturated fat	6.4 ± 0.1	5.7 ± 0.4	0.100
Use of antenatal supplements	1.7 ± 0.2	1.0 ± 0.5	0.160
HEI-SG	52.0 ± 0.6	46.9 ± 1.8	0.008

Data were drawn from the Growing Up in Singapore Towards Healthy Outcomes study and analyzed using general linear models, adjusting for maternal age, ethnicity, educational level, early pregnancy body mass index and total daily energy intake. HEI-SG, Healthy Eating Index for pregnant women in Singapore; day eaters, consumption of ≥50% of total daily energy intake between 7:00 a.m.–6:59 p.m.; night eaters, consumption of >50% of total daily energy intake between 7:00 p.m.–6:59 a.m.; SE, standard error. *p*-Value was based on independent t-test, *p* < 0.05 indicates statistical significant.

**Table 3:** Comparisons of plasma levels of micronutrients between day eaters and night eaters (*n* = 852) at 26–28 weeks gestation.

	Day Eaters (*n* = 730)	Night Eaters (*n* = 122)	
Plasma Nutrients	Mean ± SE	Mean ± SE	*p*-Value
Vitamin B6, nmol/L	78.0 ± 2.7	64.2 ± 8.0	0.107
Vitamin B12, pmol/L	299.7 ± 5.1	272.9 ± 15.2	0.126
Folate, nmol/L	17.3 ± 0.9	13.4 ± 2.7	0.010
Vitamin D (25OHD), nmol/L	78.4 ± 1.2	71.3 ± 3.7	0.025
Ferritin, ng/mL	26.1 ± 0.6	28.7 ± 1.7	0.079

Data were drawn from the Growing Up in Singapore Towards Healthy Outcomes study and analyzed using general linear models, adjusting for maternal age, ethnicity, educational level, early pregnancy body mass index and total daily energy intake. All nutrients were log-transformed for analysis. Day eaters, consumption of ≥50% of total daily energy intake between 7:00 a.m. and 6:59 p.m.; night eaters, consumption of >50% of total daily energy intake between 7:00 p.m. and 6:59 a.m.; SE, standard error; 25OHD, 25-hydroxyvitamin D. *p*-Value was based on independent t-test, *p* < 0.05 indicates statistical significant.
